# Supplementary material for: Content-rich biological network constructed by mining PubMed abstracts
Source: BMC Bioinformatics. 2004 Oct 8;5:147. doi: 10.1186/1471-2105-5-147 (PMC528731; doi:10.1186/1471-2105-5-147)
Supplement: Additional File 2 — The original results of the above study (non-essential files are deleted to keep the file size under the limit set by BMC bioinformatics). [file 1471-2105-5-147-S2.bz2 › chilibotAdditionalFile2/dip05/576ID9204896E10944/html/TEK_ANGPT2.html]

 


 **TEK** and **ANGPT2** 
  
Found 131 abstracts in PubMed, retrieved 05.  
 

 What does Google say? 
 PDF only 
| .edu only 

---

**Interactive relationship** (e.g. stimulation, inhibition, etc)

**Stimulatory relationship**- Using a combination of immunohistochemistry, laser capture microdissection and real time quantitative reverse transcription PCR, we showed that tumor derived VEGF significantly up regulated the expression of Ang 2  [ **ANGPT2** ]  in host stroma endothelial cells, resulting in markedly increased Ang 2  [ **ANGPT2** ]  Tie 2  [ **TEK** ]  mRNA copy number ratio in vivo.  Ref: 12810677 Cancer Res, 2003
**Neutral relationship**- These data provide comprehensive evidence that VEGFR 1 and 2, and angiopoietin 1, angiopoietin 2  [ **ANGPT2** ]  and Tie 2  [ **TEK** ]  interactions may be involved in the preparation of endometrium for implantation, remodelling of the maternal vasculature and trophoblast invasion during the peri implantation period in this primate species.  Ref: 12887279 Reproduction, 2003
- Recent studies have implicated the Tie2  [ **TEK** ]  tyrosine kinase receptor and its main ligands angiopoietin 1 Ang 1 and angiopoietin 2  [ **ANGPT2** ]  Ang 2  [ **ANGPT2** ]  as crucial regulators of mural cell recruitment during angiogenesis.  Ref: 12876214 J Cell SciJ Cell Sci, 2003
- CONCLUSIONS VEGF and Ang2  [ **ANGPT2** ]  Tie2  [ **TEK** ]  interactions may play an important role in the pathogenesis of ROP.  Ref: 12815197 Ophthalmic Res, 2003

**Non-interactive relationship** (e.g. studied together, co-existance, homology, etc.)

- These included familiar endothelial cell associated genes such as VEGF, VEGFR 1, VEGFR 2, angiopoietin 2  [ **ANGPT2** ] , Tie1, Tie2  [ **TEK** ] , Edg1 receptor, VE cadherin, claudin 5, connexin 37, CD31, and CD34.  Ref: 12842817 Am J Physiol Heart Circ Physiol< MedlineTA>Am J Physiol Heart Circ Physiol, 2003
- Immunoprecipitation showed phosphorylation of MPC Tie2  [ **TEK** ]  on tyrosine residues upon stimulation with Ang 1 or Ang 2  [ **ANGPT2** ] .  Ref: 12876214 J Cell SciJ Cell Sci, 2003
- mRNAs for VEGF, VEGFR 1 and VEGFR 2, angiopoietin 1, angiopoietin 2  [ **ANGPT2** ]  and their receptor Tie 2  [ **TEK** ]  were localized and quantified by in situ hybridization.  Ref: 12887279 Reproduction, 2003
- Ang2  [ **ANGPT2** ]  stained positively in 18 of 38 47.3% serial sections where Tie2  [ **TEK** ]  was present, and was also co expressed with VEGF and factor VIII.  Ref: 12815197 Ophthalmic Res, 2003
- Colocalization of tie2  [ **TEK** ] , angiopoietin 2  [ **ANGPT2** ]  and vascular endothelial growth factor in fibrovascular membrane from patients with retinopathy of prematurity.  Ref: 12815197 Ophthalmic Res, 2003
- Polyclonal antibodies specific for Ang1, Ang2  [ **ANGPT2** ] , Tie2  [ **TEK** ]  and VEGF were used for immunostaining.  Ref: 12815197 Ophthalmic Res, 2003
